# Enhancing sensitivity of lateral flow assay with application to SARS-CoV-2

**DOI:** 10.1063/5.0021842

**Published:** 2020-09-24

**Authors:** Tao Peng, Xiangpei Liu, L. Garry Adams, Girish Agarwal, Bruce Akey, Jeffrey Cirillo, Volker Deckert, Sahar Delfan, Edward Fry, Zehua Han, Philip Hemmer, George Kattawar, Moochan Kim, Ming-Che Lee, Chaoyang Lu, Jon Mogford, Reed Nessler, Ben Neuman, Xiaoyu Nie, Jianwei Pan, Jane Pryor, Navid Rajil, Yanhua Shih, Alexei Sokolov, Anatoly Svidzinsky, Dawei Wang, Zhenhuan Yi, Aleksei Zheltikov, Marlan Scully

**Affiliations:** 1 Texas A&M University, College Station, Texas 77843, USA; 2 University of Science and Technology of China, Hefei 230026, China; 3 Leibniz Institute of Photonic Technology, 07745 Jena, Germany; 4 Friedrich-Schiller-University Jena, Helmholtzweg 4, 07743 Jena, Germany; 5 Xi'an Jiaotong University, Xi'an, Shaanxi 710049, China; 6 University of Maryland, Baltimore County, Baltimore, Maryland 21250, USA; 7 Zhejiang University, Hangzhou 310027, China; 8 Moscow State University, Moscow 119992, Russia; 9 Baylor University, Waco, Texas 76798, USA; 10 Princeton University, Princeton, New Jersey 08544, USA

## Abstract

Lateral flow assay (LFA) has long been used as a biomarker detection technique. It has advantages such as low cost, rapid readout, portability, and ease of use. However, its qualitative readout process and lack of sensitivity are limiting factors. We report a photon-counting approach to accurately quantify LFAs while enhancing sensitivity. In particular, we demonstrate that the density of SARS-CoV-2 antibodies can be quantified and measured with an enhanced sensitivity using this simple laser optical analysis.

The use of the lateral flow assay (LFA) technique is widespread as an inexpensive point of care (POC) tool. Applications range from the well-known pregnancy test strip to a POC diagnostic assay for SARS-CoV-2 antibodies that can be performed in a few minutes. One widely used POC configuration for a SARS-CoV-2 exposure test is the gold nanoparticle (AuNP) ELISA-like configuration shown in Fig. 1(a).^1,2^

**FIG. 1.**
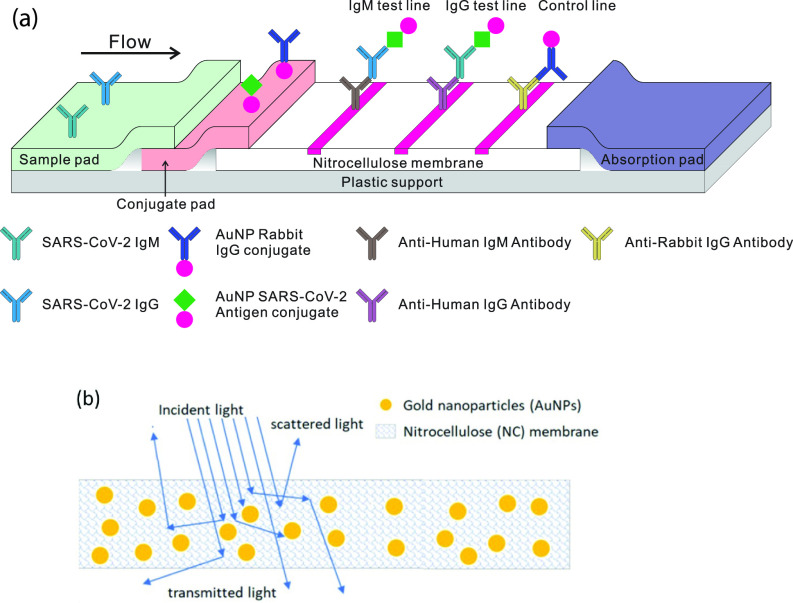
(a) Schematic illustration of a LFA showing the wicking membrane with AuNPs coated with SARS-CoV-2 spike protein. (b) Light scattering and absorption processes inside test strips with AuNPs (not to scale). The nitrocellulose membrane scatters light, and AuNPs absorb and scatter light.

These kits operate on a pinprick drop of blood and deliver a more or less binary answer to the question of infection. However, in order to detect the small amount of antibodies that accompanies an early stage infection and to make a meaningful statement about post-disease trace antibody density, a quantitative measurement with high sensitivity is required for COVID-19 surveillance and treatment^3–5^ as well as in general diagnostics.^5–7^ For example, the commercial COVID-19 test kits that we have had the best results with give a clearly visible reading in a 10 ng/ml sensitivity range as shown in Fig. 3, corresponding to about 10^11^ IgG/ml antibodies.

We would like to have a sensitivity of 0.1 ng/ml (i.e., we would like to have a test at the level of around 10^9^ IgG/ml). The questions are as follows: (1) What is the limiting feature associated with the present system? (2) What can we do to improve readout using nothing more than the present commercially existing kits? and (3) What is the limit of sensitivity? As we show in the following (see Figs. 2 and 3), a simple laser readout scheme can produce sensitivities better than 10^9^ IgG/ml, limited by nonspecific binding as discussed in the conclusion.

**FIG. 2.**
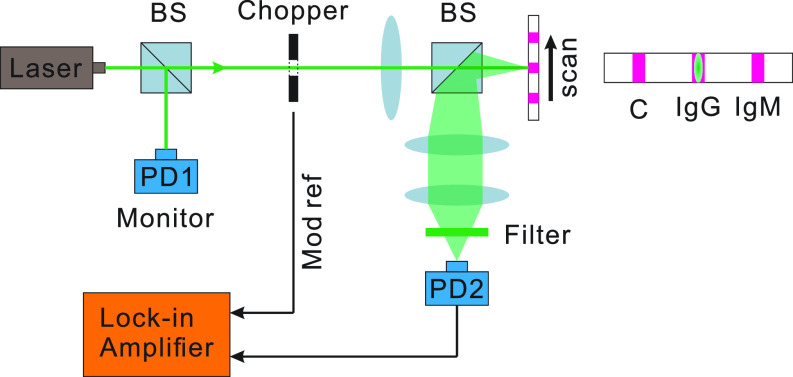
Schematic of the experiment. The 532 nm laser is split into two parts for the AuNP detection and laser power monitor. The laser vertically hits the test strip and is then collected by a photon detector through a two-lens image system.

**FIG. 3.**
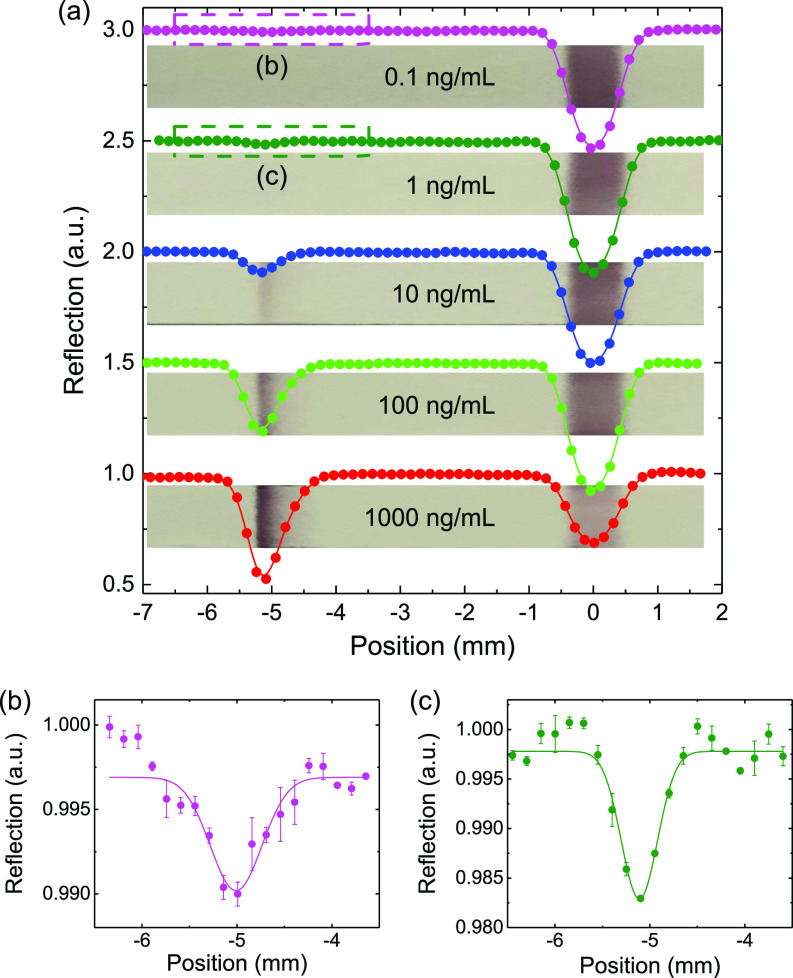
(a) Measurements on test strips with IgG concentrations from 1000 to 0.1 ng/ml, bottom to top. Three scans are taken for each sample within 5 min. Dips at ∼ −5 mm correspond to the IgG test lines, and at ∼0 mm, they are control lines. (b) and (c) correspond to densities of 0.1 ng/ml and 1 ng/ml. The solid lines are Gaussian fittings.

The main goal of the present paper is to improve the sensitivity of existing AuNP LFA test kits by improving the readout of the device without modifying any of its features. The physics of the problem will be treated in the necessary detail to accomplish this.

The fundamental problem with the process is that the nitrocellulose (NC) embeds the AuNPs in a matrix that primarily scatters the light and, thus, the reflected light is now a combination of light that has been scattered both by the NC and the AuNPs, as shown in Fig. 1(b). The problem faced by us presently is how to model both the scattering and absorption processes in the system of Fig. 1. The present paper uses the laser readout system of Fig. 2 to improve the sensitivity of the LFA test as shown in Fig. 3.

To this end, we use a 1D radiative transfer model having a simple analytical solution to be described later.^8^ The model includes spherical air voids in the NC in sufficient amount to give it a white appearance under white light illumination. To model the light scattering properties of these spherical bubbles in the NC, we will use the Lorenz–Mie theory,^9^ which is an exact solution to Maxwell's equations for a spherical scatterer and absorber embedded in a nonabsorbing medium. In order to get a simple physical picture of the LFA, we first recall the result for the reflectance of N plates stacked together. George Stokes showed that the reflectance from such a stack of plates for normal incidence is given by^10^

$$R=\frac{i_{1}}{i_{0}}=\frac{2N\rho }{1+(2N-1)\rho },$$ (1)

with reflectivity *ρ* from each surface at normal incidence. For large N, all the light is backscattered. In the present problem, we may model the fibers in the NC as playing the role of the plates. In supplementary material, we discuss the case of continuous distribution of scatterers and recover Eq. (1) for N≫1.

Next, we add the AuNP to the fiber matrix. When adsorbed on a AuNP with diameter *d* and dielectric function *ϵ_m_*, IgG/IgM forms a gold–protein conjugate coating^11,12^ of thickness *s* and with dielectric function *ϵ_p_*, changing the dielectric properties of the AuNPs–IgG/IgM–LFA composite medium and adding to the complexity of its quantitative analysis. Making the optical extinction *R* a meaningful measurable quantifier of SARS-CoV-2 is its intuitive, calibration-friendly relation to the density *N* of IgG/IgM-coated AuNPs, $R\propto \sigma_{\;\text{ext}}NL$, with $\sigma_{\;\text{ext}}$ and *L* being the extinction cross section and the beam path length. The cross section $\sigma_{\;\text{ext}}$ is generally a sum of absorption and scattering terms, $\sigma_{\;\text{ext}}=\sigma_{\;\text{abs}}+\sigma_{\;\text{sca}}$, which can be expressed in terms of the polarizability *α* of a coated sphere [Ref. 13, Eqs. (5.13)] as

$$\sigma_{\;\text{abs}}=\frac{2\pi }{\lambda }\sqrt{\epsilon_{\ell }}\;\text{Im}\;\alpha ,\;\sigma_{\;\text{sca}}=\frac{8}{3}\frac{\pi^{3}}{\lambda^{4}}\epsilon_{\ell }^{2}|\alpha {|}^{2}.$$ (2)

The polarizability of the coated sphere is known to be

$$\alpha =4\pi {\left(\frac{d}{2}+s\right)}^{3}F,$$ (3)

$$F=\frac{f(\epsilon_{m}-\epsilon_{p})(\epsilon_{\ell }+2\epsilon_{p})-(\epsilon_{\ell }-\epsilon_{p})(\epsilon_{m}+2\epsilon_{p})}{2f(\epsilon_{\ell }-\epsilon_{p})(\epsilon_{p}-\epsilon_{m})+(2\epsilon_{\ell }+\epsilon_{p})(\epsilon_{m}+2\epsilon_{p})}.$$ (4)

Here, $\epsilon_{\ell }$ is the dielectric function of the LFA in the test strip and $f=\frac{1}{{\left(1+\frac{2s}{d}\right)}^{3}}$ is the volume fraction of the protein relative to gold. We note that the polarizability of the coated nanosphere can be computed by solving electrostatic equations in a manner similar to the case of an uncoated sphere (Ref. 14, Sec. IV D). In supplementary material, we obtain formulas for the scattering and absorption cross section as a long wavelength limit of the Mie scattering functions. The cross section is expressed as a sum over multipoles. The amplitude coefficients for the field expansion over the multipole mode functions are obtained by applying the boundary conditions for the electromagnetic field at the two surfaces of dielectric discontinuity.

As the denominator of *F* approaches zero, $\sigma_{\;\text{abs}}$ rapidly grows, via surface plasmon resonance (SPR),

$$2f(\epsilon_{\ell }-\epsilon_{p})(\epsilon_{p}-\epsilon_{m})+(2\epsilon_{\ell }+\epsilon_{p})(\epsilon_{m}+2\epsilon_{p})=0,$$ (5)

leading to a resonant enhancement of the optical readout *R*.

Solving this equation for *ϵ_m_* yields the SPR condition in the form

$$\epsilon_{m}=-2\epsilon_{p}\frac{1+f\alpha_{s}}{1-2f\alpha_{s}},$$ (6)

where $\alpha_{s}=(\epsilon_{\ell }-\epsilon_{p})/(\epsilon_{p}+2\epsilon_{\ell })$.

For uncoated AuNPs, *s* = 0 and *f* = 1, Eq. (6) recovers a well-known SPR condition $\epsilon_{m}=-2\epsilon_{\ell }$. In the opposite limit of an infinitely thick coating, s≫d, setting f→0 in Eq. (6) leads to $\epsilon_{m}=-2\epsilon_{p}$. Now, the protein shell plays the role of the surrounding dielectric medium, as the nanoparticle does not even see the LFA medium through its infinitely thick coating. The SPR condition of infinitely thick coating is, thus, fully symmetric to the uncoated case with a trivial replacement $\epsilon_{\ell }\to \epsilon_{p}$.

We find that there is a resonant absorption of green photons incident on a AuNP. Hence, if we use green 532 nm laser radiation, it will be backscattered by the NC fibers but at a lower intensity due to the photons absorbed by the AuNP (see Fig. 3). This is the basis of our approach to “sense” the AuNPs at a very dilute density.

The schematic of our apparatus is shown in Fig. 2. A 532 nm CW laser is split into two beams by a non-polarizing beam splitter (BS). One part is collected by a photodetector (*PD*1) used as a laser power monitor. The transmitted laser is modulated at a 1 kHz frequency by an optical chopper then shaped into an ∼2.5 mm ×0.5 mm line and normally incident at the test strip. The substantial transverse dimension can effectively reduce the signal fluctuation from random scattering in the NC membrane. We automatically scan the strip and the scan range covers both the control line and IgG test line of the test strip. The light back-scattered by the NC will be collected onto a photodetector *PD*2. The random environmental and electrical noise is suppressed by a lock-in system. *PD*1 is used to further suppress the noise from laser power fluctuation.

We use the SARS-CoV-2 IgG (Anti-Covid 19 + SARS COV S Glycoprotein Human IgG1[CR 3022)] instead of the infectious human blood sample in the experiments. For each sample, we add the 20 *μ*l sample to the test strip sample well immediately followed by 80 *μ*l buffer. Then, a series of samples are prepared with IgG concentrations varying from 1000 to 0.1 ng/ml in a synthetic human blood plasma, where the plasma is modified based on Ref. 15 with the following additions for making up 1 l: MEM 50X essential amino acids (2%); NEAA 100X amino acids (1%); 0.9 g Glucose; 6% BSA. The pure blood plasma will serve as a background reference of the detection system. We note here that when using samples from fingerstick blood, serum and plasma will give comparable results.^1^ The pore of the sample pad in LFA can also act as a red blood cell filter.^16^ For each sample, three repetitive scans will be taken in 5 min. Figure 3(a) gives a set of measurement results. To eliminate the influence of the water on the scattering properties of the test strip, we dry each sample before the measurement. The light brown backgrounds with darker lines are actual photos of the test strips taken with a camera. The test lines are clearly visible to the eye with concentrations equal to or larger than 10 ng/ml. For lower concentrations, we can see nothing with the naked eye, while the measurement results do show a clearly measurable dip [see Figs. 3(b) and 3(c)] for concentrations of 1 ng/ml and 0.1 ng/ml. The depth of the dip near the test line is a good quantification of the AuNP density and, thus, the antibody concentration. Because the waist of the laser spot is comparable to the width of the test line, it is reasonable to fit the data with a Gaussian function $c-ae^{-{\left(x-x_{0}\right)}^{2}/w^{2}}$. We define the signal contrast as *a*/*c* to characterize the laser absorption by the AuNP.

The results for samples with different concentrations are summarized in Fig. 4. We find that the data are described by^17^

$$C=A\frac{Bn_{A}}{1+Bn_{A}}+c_{0},$$ (7)

where *C* is the contrast, *n_A_* is the concentration of the antibody, and A, B, and *c*_0_ are the fitting parameters. The shaded area indicates the measurement uncertainty for pure plasma, which sets a noise floor in our detection. This uncertainty derives from the manufacturing procedures for the test strip. Nevertheless, as shown in Table I of supplementary material, the present approach yields the lowest limit of detection (LOD) for the AuNP LFA test kits.

**FIG. 4.**
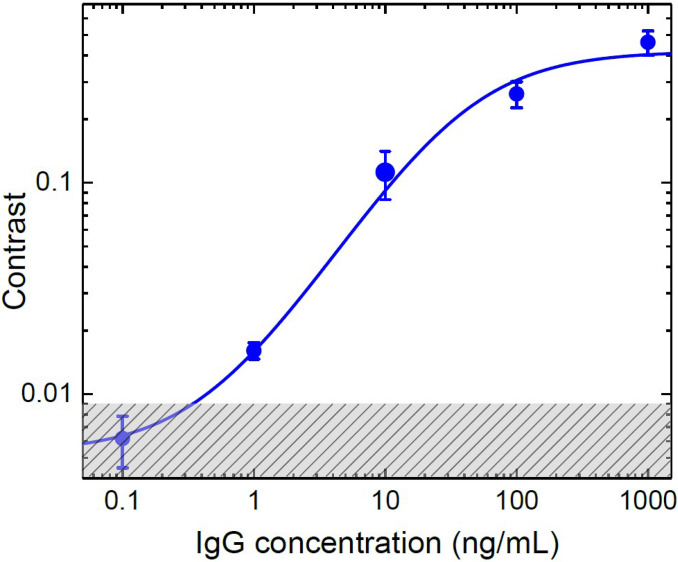
Contrasts for different IgG concentrations. 20 *μ*l solution is used for each sample. In total, four sets of samples are measured.

In summary, we theoretically analyzed the light scattering and absorption process of LFA. We then demonstrated a technique to quickly and quantitatively read out the LFA strip for SARS-CoV-2 antibodies. Compared with the traditional qualitative visual evaluation, our method not only has high specificity, low cost, simplicity of operation, and rapidity of analysis but is also capable of providing a quantification criterion for the antibodies (or the concentration of antibody in the tested sample) being detected; and it does this with high sensitivity and accuracy. This work will provide a guide for quantification of the stage of SARS-CoV-2 infection in clinical analyses.^18,19^ In particular, it opens possibilities for detecting the antibody residue after being cured of SARS-CoV-2.

See the supplementary material for the one-dimensional model of light propagating in a conservative media, scattering of light from two concentric spheres, and comparison of different quantitative LFA methods.

## AUTHORS' CONTRIBUTIONS

T. P. and X. L. contributed equally to this work.

## Data Availability

The data that support the findings of this study are available upon reasonable request.
